# A review of primary healthcare practitioners’ views about nutrition: implications for medical education

**DOI:** 10.5116/ijme.6271.3aa2

**Published:** 2022-05-26

**Authors:** Clare Carter, Joanna E. Harnett, Ines Krass, Ingrid C. Gelissen

**Affiliations:** 1 School of Pharmacy, Faculty of Medicine and Health, University of Sydney, New South Wales 2006 Australia

**Keywords:** Nutritional counselling, primary health care providers, dietary advice

## Abstract

**Objectives:**

This study aimed to review literature that reports on the perspectives and opinions of Australian and New Zealand primary healthcare practitioners on their role in nutrition counselling of their patients.

**Methods:**

A systematic search of relevant articles reporting on attitudes towards nutrition counselling by Australian and New Zealand doctors/physicians, nurses including midwives, pharmacists and dentists was conducted. The search included literature from the past ten years until March 2021 and identified 21 relevant papers, with most of the studies including medical practitioners and nurses.

**Results:**

Three main themes were identified from qualitative and quantitative data, which included education and training, practitioner experiences and challenges. Consistent with previous literature, health care practitioners acknowledged their important role in the provision of dietary advice to patients. Challenges that influenced the provision of this advice included insufficient education and training, time constraints and limited knowledge and confidence. Time constraints during normal consultations led to a low priority of nutrition counselling. An absence of assessment opportunities to demonstrate nutrition competence and limited coverage of specific nutrition-related advice during training were also reported.

**Conclusions:**

Primary healthcare practitioners acknowledge the importance of playing a role in the provision of nutrition advice but require education and access to evidence-based information that can be utilised effectively within the time constraints of standard consultations. Medical education curricula can be improved to provide more emphasis on nutrition education, including relevant assessment opportunities.

## Introduction

The World Health Organisation (WHO) describes nutrition as a ‘fundamental pillar of human life, health and development across the entire life span’.^1^ The rise in poor dietary habits, underpinned by the consumption of energy-dense foods high in saturated and trans-fats, refined sugars, and excess salt, has precipitated a worldwide epidemic of non-communicable diseases.^2^ Nutrition is a key modifiable determinant of non-communicable diseases, for which evidence illustrates the impact of changing dietary patterns on health outcomes.^2^ More specifically, dietary interventions play a crucial role in the prevention and treatment strategy of chronic diseases, including diabetes, cardiovascular disease and hypertension.^2^ In 2017, dietary risk factors accounted for 11 million deaths globally.^3^

In addition to the valuable role of dieticians, who are instrumental in the education of patients with existing chronic diseases, it has been recognised worldwide that primary healthcare practitioners can also play a fundamental role in the provision of evidence-based nutrition information to patients.^4^ For the purpose of this study, the term ‘primary healthcare practitioners’ describes medical doctors/physicians, pharmacists, nurses and/or dentists. Primary healthcare practitioners are regarded as a relatively large, affordable, and accessible community for whom the implementation of strategies to guide the provision of nutrition care could be advantageous. This is apparent in rural settings where access to dieticians may be limited.^5^^,^^6^ An understanding about the knowledge, skills and attitudes of primary healthcare practitioners towards their role in the promotion of healthy nutrition is warranted.

While several studies have reported healthcare practitioners’ perceptions about dietary counselling, a comprehensive review of literature including primary healthcare practitioners of Australia and New Zealand has previously not been conducted.^7^^, ^^8^ Therefore, the aim of this review was to identify and summarise the current literature that documents primary healthcare practitioners’ self-perceived knowledge and opinions about the role and readiness to counsel patients on healthy nutrition. The findings of this review will inform the development of education initiatives that aim to equip primary healthcare practitioners with the knowledge and skills required to provide dietary counselling to their patients.

## Methods

A systematic search and review, as described by Grant and Booth was conducted to identify and summarise peer-reviewed literature that reported perspectives of Australian and New Zealand primary healthcare practitioners about their knowledge and readiness to counsel patients in nutrition.^9^ Australia and New Zealand were chosen due to socio-demographic similarities of the populations and similarities in the training of primary health care practitioners.

### Search Strategy

Medline, Embase, Web of Science and Scopus were searched for key concepts related to nutrition and primary healthcare practitioners’ provision of dietary counselling. Google Scholar was also searched to capture any articles not identified in the main search. The search was conducted between 19th March and 9th April 2021. The inclusion/exclusion criteria were agreed upon by three authors, and a University of Sydney Faculty of Medicine and Health librarian was consulted regarding the search strategy. The following studies were included: original research studies conducted in Australia and New Zealand adults, written in English, reporting on primary healthcare practitioners’ opinions about dietary counselling, involving doctors/physicians, nurses (including midwives), pharmacists and dentists. Only articles from the past ten years were included to align as much as possible with the most recent curricula provided to health care practitioners. Studies were excluded if they involved allied healthcare professionals, including dieticians, physiotherapists, occupational therapists, chiropractors, naturopaths and complementary medicine practitioners (e.g., herbalists) and complementary medicine products. In addition, any studies involving participants who were students were also excluded. Furthermore, review articles, books, policy documents and conference proceedings were excluded, as were articles in languages other than English or those involving countries other than Australia and New Zealand. Search results were uploaded into EndNote, and duplicates were removed. Titles and abstracts were screened by one author (C.C.) and checked by two additional authors (I.G. and J.H.). Full-text articles included in the study were screened by three authors (C.C., I.G. and J.H.) to confirm eligibility and extraction of relevant data as outlined below. A PRISMA flow diagram of the literature search methodology is included in Appendix 1.

### Data extraction and analysis

Data were extracted, summarised, and tabulated using author, year of publication, aims, study method, sample population and sampling methods, as well as key findings and outcomes (Table 1). As the survey tools utilised in the studies varied substantially, including qualitative data generated from interviews and focus groups, further analysis was performed to assist in the interpretation and organisation of data, utilising the six phases of analysis by Braun and Clarke.^10^ This included initial data familiarisation and key concept identification by one author (C.C.), followed by coding and identification of meta themes and sub-themes agreed upon by three authors (C.C., I.G. and J.H.). Discussions throughout the analysis process allowed for consensus between members of the research team regarding the interpretation of data, theme conceptualisation and naming.

## Results

A total of 520 articles were identified in the literature search, with an additional article retrieved through Google Scholar. Following the title and abstract screening of 382 articles, 30 articles were assessed for suitability against our inclusion criteria and critically evaluated, with a total of 21 articles included in the systematic review.

An overview of the studies is presented in Table 1, including the aim, the population studied, the methods utilised, and the major findings reported by the authors. With regards to the primary healthcare practitioners sampled in the articles, the following breakdown of healthcare practitioners was found: general practitioners (n=10), general practitioner registrars (n=3), general practitioner interns (n=1), general nurses (n=10), midwives (n=2) and pharmacists (n=1). Participant numbers averaged 125 (range 9 – 393), with 9 out of 21 studies having >100 participants. Study methods included quantitative methods (questionnaires and surveys; n=16 with a mix of paper and online delivery), qualitative methods (semi-structured interviews n=4; focus groups; n=1) and mixed methods (n=1). The predominant sampling method was convenience sampling. The topics covered included general nutrition (n=7), pregnancy (n=5), nutrition and chronic conditions (including type II diabetes; n=4), nutrition and cancer (n=2), and more specialist branches of nutrition (enteral nutrition, malnutrition and dehydration, and nutrition for patients with brain injuries; n=3).

**Figure 1:**
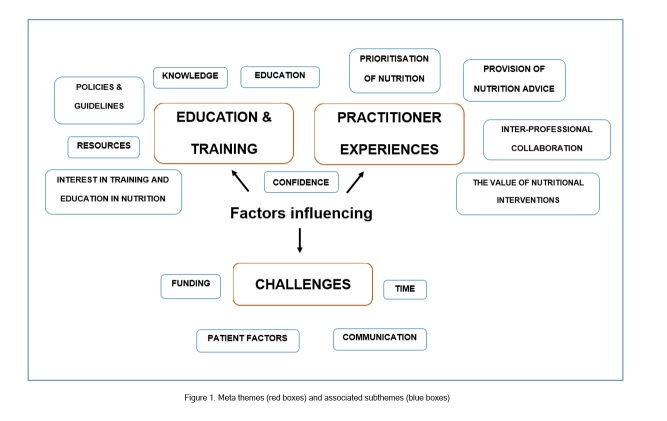
Meta themes (red boxes) and associated subthemes (blue boxes)

After coding of articles, three meta themes and fourteen subthemes were identified from the qualitative data as well as the topics and outcomes from the quantitative surveys and questionnaire data. Figure 1 illustrates the meta themes, which were 1) education and training, 2) practitioner experiences and 3) challenges, presented in red, with associated sub-themes in blue. Table 2 provides an overview of selected quotes, supporting the meta themes and sub-themes. The quotes were extracted from individual references and reflected the perspectives of the study participants.

**Table 1:** Overview of the studies, including aims, methods and their key findings

Study	Aims	Method	Sample included	Key findings
Arrish et al. (2016)^11^	Investigate midwifes’ nutrition knowledge, confidence and attitudes regarding providing nutrition education in pregnancy	Cross-sectional online survey	329 Midwives (Australia); Convenience sampling	75.7% (n = 249) of Australian midwives recognised their role in delivering nutritional advice to pregnant women as highly significant. Midwives’ confidence ranged from moderate to low when discussing general and specific nutrition advice to pregnant women. Inadequate nutritional knowledge was recorded in several areas. Only half of the midwives (51.1%) indicated that they received nutrition education during midwives training or after registration (54.1%).
Arrish et al. (2017)^12^	Explore midwives’ nutritional knowledge gained during midwifery education and following registration, and their perceived readiness to provide nutrition counselling	Cross sectional written and online survey	393 Midwives (Australia); Convenience sampling	79.3% (n = 261) of midwives reported receiving some nutrition education during, before or after registration with many describing this coverage as limited. 94.2% (n = 310) of midwives indicated that they would benefit from receiving additional nutrition information and tailored guidelines for the provision of nutrition advice (87.8%, n = 289). A further 59.1% (n = 78) indicated that additional nutrition education would improve their knowledge of current evidence-based advice and increase their confidence.
Cass et al. (2014)^8^	Investigate practice nurses’ perceptions of their role and competency in the provision of nutrition advice to patients with chronic disease	Semi-structured telephone interview	20 Practice nurses (Australia); Purposive sampling	Lack of confidence, nutrition knowledge, time and unenthusiastic patient attitude towards nutrition influenced the provision of nutrition care to patients with chronic disease. Practice nurses recognised the significance of their role in promoting basic nutrition care, but the interpretation of basic varied. Practice nurses were concerned about the lack of accessibility and availability of current nutrition education opportunities.
Chapple et al. (2018)^13^	Explore the attitudes, experiences and barriers of healthcare practitioners that influence their decisions in providing nutritional therapy to patients with traumatic brain injuries (TBI)	Semi-structured interview	18 Nurses 16 Physicians (Australia); Purposive sampling	Health practitioners were unclear about the role they played in the provision of nutrition therapy to TBI patients, hence there was a lack of perceived responsibility in the management of nutrition. There were competing priorities when caring for patients with TBI, identifying a need for further education of the multidisciplinary team to gain an understanding of current evidence-based guidelines to enhance nutrition practices in the TBI population.
Crowley et al. (2016a)^7^	Identify and describe general practitioners’ interest, confidence and barriers in the promotion of nutrition care	Cross-sectional online and written survey	322 GP’s* (Australia); Convenience sampling	A large proportion of general practitioners (91.6%, n = 295) were very interested and 71.7% (n = 231) reported moderate confidence in providing nutrition care to patients where long-term strategies were well-established. General practitioners documented time constraints as the biggest barrier in the provision of nutrition care during consultations (52.8%, n = 170). An overwhelming majority of general practitioners were interested in undertaking additional education and training to improve nutritional knowledge and skills. The study concluded that general practitioners would benefit from educational programmes that focused on the delivery of healthy dietary practices within standard consultations and identification of nutritional risk.
Crowley et al. (2015)^14^	Describe general practitioner registrars and general practitioners perceived attitudes and skills in the provision of nutritional advice	Cross-sectional paper-based questionnaire	51 GP registrars 57 General practitioners** (New Zealand); Convenience sampling	General practitioners and general practitioner registrars reported positive attitudes towards the incorporation of nutrition care in consultation with patients. Lack of confidence in nutritional skills such as the role of food constituents in health and the basic metabolic role of protein, carbohydrates and fats was documented. An enhancement of knowledge in this area was identified as beneficial in future.
Crowley et al. (2016b)^15^	Explore the perceptions of general practitioners in the promotion of nutrition care to patients with chronic disease	Focus groups	48 GP’s (New Zealand); Convenience sampling	Limited consultation time restricted nutritional competence while patients’ resistance to change restricted general practitioners in providing basic nutrition care to patients with chronic disease. General practitioners reported receiving inadequate education during medical training and expressed the need for further information to provide economically, socially and culturally sensitive nutrition care.
Crowley et al. (2016c)^16^	Investigate the ability of general practitioner registrars to counsel patients on nutrition	Cross-sectional survey	47 GP registrars (New Zealand); Convenience sampling	General practitioner registrars recorded a positive attitude towards the implementation of nutrition care but only moderate confidence in the implementation of this care. A limited number of general practitioner registrars provided evidence-based nutrition advice which was identified as potentially due to gaps in their nutrition knowledge. Lack of experience, lack of assessment opportunities, attitude and awareness of nutrition guidelines were considered factors that may influence the quality of nutrition care provided by general practitioner registrars.
El-Mani et al. (2014)^17^	Assess the knowledge of pharmacists regarding folic acid and iodine supplementation during pregnancy	Cross-sectional survey	41 Pharmacists (Australia); Convenience sample	Pharmacists’ knowledge regarding the mandatory fortification program was limited as only 46% (n = 19) and 68% (n = 28) correctly identified that bread must be fortified with folic acid and iodine respectively in Australia. 49% (n = 20) of pharmacists selected the correct dietary sources of folic acid, but only 12% (n = 5) correctly identified dietary sources of iodine. In order to enhance pharmacists’ knowledge of current evidence-based nutrition guidelines in pregnancy, additional education of pharmacists was identified as needed.
Fieldwick et al. (2019)^18^	Explore general practitioners’ knowledge and practice regarding gestational weight management	Survey	200 GP’s (New Zealand); Cluster/Random sampling	Sixty-six general practitioners reported always discussing nutrition during pregnancy-related consultations. The knowledge and practice of general practitioners regarding gestational weight gain was not in accordance with national guidelines. Twenty-three general practitioners identified a lack of time and 19 suggested financial stress as barriers to providing all necessary nutritional advice.
Forsyth et al. (2012)^19^	Evaluate the confidence and knowledge of nurses in the provision of evidence-based practice advice to patients in a forensic psychiatry rehabilitation unit	Semi-structured interviews	9 Nurses (New Zealand); Convenience sampling	Although they recalled having basic nutritional knowledge, nurses felt that they had low confidence in the provision of simple nutritional information to patients. Lack of formal nutrition education amongst nurses, and patient’s resistance to change were key barriers preventing the promotion of nutritional changes. An evaluation of nutrition education needs of nurses was identified as necessary to enhance the promotion of nutritional practices to patients.
Lucas et al. (2014)^20^	Assess the nutritional knowledge and practices of pregnant women and healthcare providers who participate in antenatal shared care	Survey	50 GP’s 11 Nurses (Australia); Convenience sampling	General practitioners and nurses had poor knowledge about the importance of iodine and the role of iodine in pregnancy. Most healthcare practitioners reported interest in undertaking additional education training or education regarding iodine in pregnancy.
Martin et al. (2014)^21^	Investigate the opinions of practice nurses on the promotion of nutrition care in chronic disease management	Cross-sectional online survey	181 Practice nurses (Australia); Convenience sampling	A large proportion (89%, n = 143) of practice nurses understood the significance of their role in addressing nutrition behaviours of patients, but 61% (n = 95) were uncertain whether their nutrition counselling was effective in improving the nutrition behaviour of patients. 53% (n = 85) of practice nurses agreed/strongly agreed that practice nurses do not have the adequate training to discuss nutrition with patients, and most desired further education. Insufficient time restricted practice nurses in providing nutrition care to every patient.
Mitchell et al. (2011)^22^	Discuss general practitioners and practice nurses’ role in the delivery of nutritional information to patients in a primary health setting	Mixed methods cross-sectional study: questionnaire, semi-structured telephone interview and online survey	10 GP’s 12 Practice nurses (Australia); Convenience sampling	A large proportion of general practitioners (90%, n = 9) and practice nurses (83.3%, n = 10) agreed/strongly agreed that dietary assessment and counselling was integral to their professional role. Most general practitioners (n = 6) and practice nurses (n = 6) disagreed that they had enough time to provide nutrition advice. Further nutrition education, availability of resources and nutrition-related guidelines was identified as required to effectively provide nutritional advice.
Morphet et al. (2016)^23^	Explore nurses’ enteral nutrition knowledge and sources of information	Online questionnaire	359 Nurses (Australia); Convenience sampling	Most nurses rated their knowledge of enteral nutrition as good (60.1%, n = 205) or excellent (10.3%, n = 35). Knowledge deficits were identified in the following areas: gut physiology, malnutrition, feed formulation, and administration rates. Respondents recalled receiving very little enteral nutrition education during professional nursing training. 272 respondents (or 75.8%) indicated that they wanted further education regarding enteral nutrition, and many identified the need for increased access and availability of enteral nutrition guidelines and policies.
Nowson and O’Connell (2015)^24^	Assess general practitioner registrars’ knowledge, confidence and perceived role regarding the provision of nutrition advice	Online survey	93 GP registrars (Australia); Convenience sampling	Approximately half of general practitioner registrars (51%) felt moderately confident and 16% very confident in their ability to provide nutrition advice. All participants agreed that they have a role in the provision of nutrition care to patients. Additionally, although most of the participants recalled getting nutrition information during training (84%), only 34% recalled having to demonstrate their nutritional knowledge during their training. Overall, general practitioner registrars lacked consensus with regards to their role in providing nutritional advice.
Parry Strong et al. (2014)^25^	Assess the knowledge, skills and resources of practice nurses used to advocate nutrition advice to patients with type 2 diabetes	Questionnaire	113 Practice nurses (New Zealand); Convenience sampling	The confidence of practice nurses in the counselling of dietary behaviours to people with type 2 diabetes decreased from 83% to 70% between 2007 and 2012. Across surveys, dieticians were the most sought-after healthcare professionals for practice nurses seeking advice beyond their own nutrition knowledge. Future training sessions that address culturally specific dietary advice and quick nutritional assessment skills are warranted.
Puhringer et al. (2015)^26^	Investigate the nutrition promotion practices, beliefs and barrier of cancer nurses	Online questionnaire	123 Cancer nurses (Australia and New Zealand); Convenience sampling	A proportion of cancer nurses (35%, n = 43) identified dieticians as the primary source of the provision of nutrition care to patients, while an equal proportion (32.5%, n = 40) regarded themselves as the primary healthcare professionals that addressed the nutritional requirements of cancer patients. Most nurses agreed/strongly agreed that healthy eating improves health-related quality of life. Lack of time and nutrition expertise were cited as the most common barriers to promoting healthy eating.
Waterland et al. (2020)^27^	Report general practitioners’ experiences, barriers and enablers regarding providing nutrition and exercise advice to cancer patients	Semi-structured telephone interviews	33 GP’s (Australia); Purposive sampling	General practitioners acknowledged the importance of their role in the ongoing promotion of nutrition and exercise counselling in patients with cancer. General practitioners noted the influence of patients’ attitudes, insufficient time, and the lack of resources and programs on nutrition advice and exercise practices. As general practitioners expressed feeling underqualified to counsel cancer patients in these areas, a strong desire for additional education and training was evident.
Whitelock and Kapur (2018)^28^	Determine the knowledge, practices and attitudes of interns about malnutrition and hydration in an acute tertiary-care hospital	Questionnaire	34 GP registrars (Australia); Convenience sampling	General practitioner interns recorded poor knowledge of the principles regarding malnutrition and hydration management. Overall, almost all interns agreed that further training in malnutrition (90%, n = 76) and hydration (88%, n = 74) would be beneficial and improve the health outcomes of patients. Competing priorities, lack of interest and unclear sense of responsibility were identified as barriers that restricted the provision of nutritional advice to patients.
Winter et al. (2017)^29^	Understand general practitioners and practice nurses’ experiences and current practices regarding the nutrition care of patients	Online survey/ questionnaire	45 Practice nurses and GP’s (Australia); Convenience sampling	63% (n = 24) of practice nurses and general practitioners felt moderately to very confident in their ability to promote nutritional recommendations. Despite high perceived confidence, 68% (n = 26) indicated the need for further professional development in this area. Inadequate understanding of knowledge and the absence of guidelines were regarded as challenges to the provision of nutritional information.

*GP = General Practitioner; **Articles also investigated other population samples, including primary healthcare practitioners from other countries, allied health practitioners, students, and the general population. These findings were excluded from our analyses.

**Table 2:** Illustrative quotes by participants relevant to meta themes and subthemes

1. PRACTITIONER EXPERIENCES	
	1.1 Prioritisation of nutrition (n = 4 articles)
	‘The ideal role is that if you’re not taking the opportunity to talk to patients about their diet and exercise, you are just missing every opportunity to save the nation’s health in every way possible’.^8^
	‘Diet and exercise are part of the consultation. It’s routine’.^15^
	‘General practitioners have a very important role in providing this [nutrition] advice’.^24^
	‘[I]t is our role as general practitioners to enquire about [exercise and nutrition] and provide support as needed’.^27^
	1.2 Inter-Professional collaboration (n = 4 articles)
	‘Currently unable to refer women to [the] dietician, [due to] (funding)…therefore midwives, especially those involved in continuity of care models are at the coal face to make changes given they have the right information and tools to do so’.^12^
	‘Obviously it needs to be a joint effort between the doctors and the nursing staff and the dietician’.^13^
	‘It would be helpful to have dietician provide in-service with select critical care scenarios to gain understanding for how she approaches a patient case and chooses dietary formula based on patient’s past history, multiorgan failure, type of surgery done, allergies, etc’.^23^
	‘I haven’t referred to exercise physiologists because there aren’t that many around and the referral pathways are not well developed in our region. I’ve certainly referred to physios but usually on an EPC [enhanced primary care] type thing and that’s not enough. They need more’.^27^
	1.3 The value of nutritional interventions (n = 3 articles)
	‘I preferred not to answer the Q [question] about how many serves of dairy to give adequate calcium – because I don’t believe dairy is healthy for anyone’.^11^
	‘I’m not aware of any studies…which have actually made a difference to the outcome of these patients’.^13^
	‘Probably better than any medication that we can give you’.^27^
	1.4 Prioritisation of nutrition (n = 2 articles)
	‘It’s [nutrition] an important thing, but it’s not an essential…like the airway, breathing, circulation…those things will always come first’.^13^
	‘…dealing with acute, immediate issues that the patient has come in with…then not getting the chance to talk about things like exercise and nutrition’.^27^
2. EDUCATION AND TRAINING	
	2.1 Knowledge (n = 4 articles)
	‘I’m sure there’s research in that area [nutrition interventions], but I don’t know anything about it’.^13^
	‘Lack of knowledge, which stems from lack of training. You learn from what you read in the patient information sheets’.^15^
	‘I know very little about the management of weight in pregnancy and the implication of obesity…’.^18^
	‘I do a lot of background readings in this idea [nutrition counselling] so I feel like I have a lot of knowledge on how to work through that but I don’t think this is a common thing at all in general practice’.^27^
	2.2 Confidence (n = 3 articles)
	‘To be honest I wouldn’t feel 100% confident. I mean I have certain knowledge towards it [nutrition] but I don’t feel very confident’.^19^
	‘I would like to build on knowledge to gain confidence’.^25^
	‘[It’s] as important as all the pharmacological treatment but probably as [general practitioners] we don’t do it enough and don’t have enough confidence in giving recommendations regarding exercise and nutrition’.^27^
	2.3 Interest in training and education in nutrition (n = 6 articles)
	‘Midwives are often the first point of contact a mother has with a health professional during her pregnancy and should be given every opportunity to expand knowledge and give the best advice to mothers and their families’.^12^
	‘I think we [practitioner nurses] should all do a nutritional course, solely on nutrition…’.^8^
	‘As health professionals we can help with the guidelines… we are not trained. Going on a course of or having a CE session does not make you an expert. Basic stuff is ok, but then flick on’.^15^
	‘Would like further education re re-feeding syndrome’.^23^
	‘I need to have more education and training on this [nutrition] to do [counsel] it properly’.^25^
	‘I think having resources that are simple and easy to use that are fairly generic so that [they] can be used for most cancers and a handout for patients would be incredibly useful’.^27^
	2.4 Education (n = 3 articles)
	‘…and you know we don’t really have enough staff education before people come in or when they come in. Its fly by the seat of your pants for a lot of it…’.^19^
	‘Postgraduate education provides limited education regarding nutritional therapy…’.^23^
	‘I think most medical school training needs to increase the emphasis on nutrition and addressing lifestyle risk factors’.^24^
	2.5 Policy and Guidelines (n = 3 articles)
	‘Things work so much better when we eliminate some of the shades of grey…a consistent guideline would be terrific in many areas of maternity care, not just nutrition advice’.^12^
	‘My knowledge [of enteral nutrition] was gained mostly by self-education…When I came to this ICU, the feeding policy was non-existent and feeding practices were poor’.^23^
	‘And ongoing support, continuous professional development in nutrition, I have never, I don’t think, been to one; I don’t think they’re around’.^27^
	2.6 Resources (n = 2 articles)
	‘Very little education or resources are directed towards midwives in this area and we are the health professionals who spend most time with the women’.^12^
	‘I think that’s the real issue; I don’t think I have a real resource, I’m just using my brain, my common sense. I don’t think I’ve ever had any tuition about diet and cancer’.^27^
3 CHALLENGES	
	3.1 Time (n = 5 articles)
	‘We [practitioner nurses] are just so busy and just so overworked that we don’t get time to do that sort of thing [provision of nutrition care]’.^8^
	‘It is good to talk about nutrition, but how do you fit that in when people come in with an agenda for a 15-minute consult?’.^15^
	‘15 minutes appointment for first pregnancy (the only one I see) is barely enough time to discuss folic acid + iodine, midwife referral, listeria… so [I] don’t often also discuss weight’.^19^
	‘…I feel time is a large impediment in the general practitioners’ ability to provide complex nutritional advice to patient’.^24^
	‘Ten-minute consultations are simply spinning the wheels in the mud. You can’t do anything, because you don’t have the time to do anything’.^27^
	3.2 Patient factors (n = 5 articles)
	‘We are seeing more obese women and it is difficult to give then advice on weight management during pregnancy’.^12^
	‘Patients have their own agenda. They come with their own list. They don’t want the general practitioner talking about subjects not relevant to the list’.^15^
	‘Well they don’t really ask us anything, they try not to because they don’t want to be told not to do things they want to do. They try to get away with stuff’.^19^
	‘Someone who lacks motivation and has no other support outside their general practitioner is unlikely to succeed in a lifestyle change’.^24^
	‘Patients have their own agenda when they come to the doctor…the top of their priority list is not always to hear about nutrition, exercise and lifestyle…but you still try to weave it in’.^27^
	3.3 Funding (n = 2 articles)
	‘Under the current unsatisfactory system, including the paucity of remuneration for general practice in obstetric care, involvement [of gestational weight management] in obstetric care is impossible’.^18^
	‘Yeah, the fact that [general practitioners] don’t get funded for spending a lot of time with patients [to discuss exercise and nutrition]’.^27^
	3.4 Communication (n = 1 articles)
	‘Communication skills. To be able to talk to the patients and gather information you need. Much the same as what we’re doing now’.^8^

## Discussion

This review provides a comprehensive overview of primary healthcare practitioners’ perspectives about the counselling of patients in nutrition in Australia and New Zealand. The results illuminate several key factors that influence the opinions of primary healthcare practitioners regarding the provision of nutrition advice to patients. Firstly, primary healthcare practitioners clearly perceive the provision of nutrition advice as their responsibility, with only one article including general practitioners reporting that the provision of detailed nutrition counselling was not considered part of their role.^7^ This is consistent with previous literature, suggesting that healthcare professionals should conduct a nutritional assessment, provide basic evidence-based nutrition advice, and refer patients to a dietician when necessary.^4^^,^^30^^-^^32^ As healthcare practitioners are accessible to a large proportion of the population, this provides opportunities to discuss nutrition, encourage dietary changes and support the long-term maintenance of these dietary changes.^8^^,^^22^ However, uncertainty regarding an operational definition of basic nutrition care was reported by some primary healthcare practitioners, who found it difficult to differentiate their professional roles.^8^^,^^13^^,^^18^ Clarification of the scope of practice of healthcare practitioners in relation to nutrition may relieve uncertainty and enhance confidence in the delivery of such information.^21^ Professional associations could consider the development of a position statement or guiding principles to achieve this outcome.

Secondly, a recurrent sub-theme across the studies reviewed was the perceived value and impact of primary healthcare practitioners on patient health outcomes.^18^^,^^22^^,^^26^ Perspectives were positive about the impact of dietary counselling on changing patients’ eating patterns, where healthcare practitioners strongly agreed that nutrition is a key determinant of health outcomes.^18^^,^^22^^,^^26^^,^^27^ This concurs with the view of the WHO that the provision of nutrition services is associated with improved maternal, infant, and child health, a lower risk of chronic disease and improved life expectancy.^33^ Recognition of the crucial role of dietary interventions in the prevention and treatment of non-communicable diseases can influence the frequency of dietary counselling by primary healthcare practitioners.^8^ However, while nurses reported that they believed there is considerable evidence to support the success of dietary interventions, the strength or extent of this evidence was unknown to them^.26^ The lack of awareness of primary healthcare practitioners, prompted by a deficiency in nutrition knowledge, can precipitate negative beliefs about the effectiveness of nutrition interventions.^13^ Health professionals, particularly doctors, are shown to influence patients’ nutrient intake; hence there is a need for healthcare practitioners to lead and promote a collaborative nutrition care approach.^34^ This may be achieved by ensuring all healthcare practitioners adopt the view that improving their patients’ nutritional habits will improve patient outcomes.^34^ Additionally, the development of objective outcome measures to quantify the benefits of nutrition therapy may modify practitioners’ perspectives and help in this matter.^13^

Despite the importance of nutrition and its role in the provision of advice to patients, primary healthcare practitioners were often not able to translate this priority into practice due to a lack of education and training, with a clear gap in knowledge identified across several areas of nutrition.^8^^,^^13^^,^^15^^,^^35^ This may lead to the provision by practitioners of nutrition education based on personal experiences and perspectives rather than evidence-based guidelines, which can be of concern.^13^^,^^35^ It was also apparent that primary healthcare practitioners’ knowledge deficits impede their confidence in the delivery of nutrition information to patients.^25^^,^^28^^,^^29^ Lack of evidence-based nutrition knowledge and associated confidence is underpinned by inadequate nutrition education during and after their formal training.^15^^,^^17^^,^^23^^,^^24^ This lack of confidence was also noted in a study from Germany, where less than half of general practitioners surveyed believed that they had successfully changed the dietary habits of their patients.^36^ Specifically, studies included in this review emphasised an absence of assessment opportunities to demonstrate nutrition competence and limited coverage of specific nutrition-related advice during training.^12^^,^^15^ Following registration, there is often limited availability and accessibility of nutrition education opportunities and resources. It is therefore unsurprising, that another strong theme identified in this review was primary healthcare practitioners’ interest in receiving additional education and training in nutrition.^8^^,^^20^^,^^21^^,^^29^ This concurs with the findings of previous studies that identified insufficient education in nutrition, including a lack of nutrition assessment skills that translate into clinical practice.^30^^,^^35^^,^^37^ Implementation of a comprehensive nutrition curricula into existing Australian and New Zealand curricula and an increase in available educational opportunities and resources is clearly warranted. In Australia, a Nutrition Competency Framework was developed in 2016 to guide the inclusion of several knowledge and skills-based nutrition competencies in Australian medical curricula. This Framework has, however, to our knowledge, not been formally adopted by the Australian Medical Council, which regulates the Australian and New Zealand medical education. In New Zealand, a nutrition syllabus was introduced in 2012 in general practitioners' training. However, the effectiveness of this syllabus remains to be assessed.^38^^,^^39^ A recent comparative analysis of nutrition incorporated into medical curricula worldwide showed that only 44% of the Australia and New Zealand accreditation documents included in the study had requirements for nutrition education.^40^ A systematic review of worldwide literature on the provision of nutrition education to medical students also identified a lack of consensus in education worldwide and concluded that medical students are not provided with adequate nutrition training, calling for institutional commitment to improve nutrition education.^41^ Clearly, there is scope to improve nutrition education of medical practitioners worldwide, including assessment opportunities.

The capacity of primary healthcare practitioners to guide and provide evidence-based information to patients is further impacted by additional challenges, including time constraints, funding and prioritisation of nutrition. In Australia and New Zealand, primary health practitioners consistently reported that the time length of patient consultations highly influenced their decision to counsel patients on nutrition.^7^^,^^8^^,^^25^^-^^27^^,^^35^ For example, practitioner nurses and general practitioners indicated that 5-10 minutes and 1-5 minutes respectively were spent on discussions on patients’ diet and the provision of nutrition advice.^22^ Literature has stressed that building rapport and getting an understanding of the psycho-social requirements of patients is needed to motivate dietary changes of patients – a process that takes significantly longer than a typical 15-minute healthcare practitioner appointment.^37^ Time constraints are clearly a major challenge to the provision of nutrition advice; however, this is not a challenge that can be easily addressed as it would require budgets allocated to the provision of healthcare to include funding time for nutrition assessment and recommendations in consultations with patients.^42^ Thus, funding is an additional barrier to the implementation of nutrition advice during counselling as healthcare practitioners are not adequately reimbursed.^8^^,^^22^^,^^27^ Supplementary solutions for current time constraints include improved education concerning the delivery of brief interventions to promote healthy dietary behaviour change within the context of limited time or the collaboration with other staff that may increase practitioners’ time with patients.^21^ The challenge of prioritisation of nutrition is linked to the limited time in standard consultations with patients.^13^^,^^27^^,^^28^^,^^35^ Whilst increasing practitioners’ knowledge about the importance of nutrition interventions may encourage the provision of nutrition advice, the matter of time continues to lower the priority of nutrition over acute health-related problems.

Although many studies of the perspectives of nurses and doctors were identified, no articles reported on the opinions of dentists towards nutrition counselling. This is an unexpected finding as poor dietary habits are associated with poor dental outcomes, including cavities and gum disease, and poor dental conditions limit individuals food choices.^43^ Dental practitioners recognise the essential role of dietary counselling in the prevention of cavities, but infrequently provide brief and non-specific nutrition advice due to a perceived low level of confidence and competence.^43^ The literature also reported various challenges, including financial compensation, insufficient education and training, and time constraints.^44^ These findings coincide with the perspectives of other primary healthcare practitioners included in this review. Furthermore, only one study included in this review explored the perspectives of pharmacists toward nutrition and dietarycounselling.^17^ The Pharmaceutical Society of Australia’s National Competency Standard Framework indicates that the role of pharmacists encompasses the promotion of dietary recommendations that complements the provision of medications.^45^ In previous studies, it has been identified that pharmacists’ lack of knowledge and expertise in nutrition was a major limitation in providing dietary counselling.^46^^,^^47^ Additionally, pharmacists strongly agreed that they are an accessible and credible source of nutrition information for patients but reported low confidence in providing this nutrition-based therapy.^48^ Hence the perspectives of pharmacists and dentists align with the perspectives of primary healthcare practitioners explored in this review.

Although this study exclusively analysed the opinions of primary healthcare practitioners, dieticians are clearly specialists in providing detailed dietary advice. According to the Australian Government Department of Health, if an individual is diagnosed with a disease where nutrition plays an important role in disease management (e.g., cardiovascular disease or diabetes), general practitioners can provide a General Practitioner Management Plan (GPMP) and Team Care Arrangement (TCA), which entitles the individual to five visits per year to a registered dietician.^49^ However, patient access to dieticians may be limited due to socioeconomic factors, remote location, or patient’s health conditions not qualifying them for this rebate. In addition, the GPMP and TCA do not cover the entire cost of appointments, with out-of-pocket gaps having to be paid by patients.^49^ Previous reports highlight a shortage of dieticians in rural and regional areas where populations often demonstrate the greatest need for dietary interventions.^6^ Limitations that reduce access of patients to dieticians also include the low rate of referrals by practitioners such as physicians related to patient resistance given the cost of dietician services.^50^ Lastly, the role of nutrition in disease prevention is clearly as important as the role of dietary changes after patients are diagnosed with a chronic condition. Therefore, there is an increased obligation of primary healthcare practitioners to provide nutrition advice given their feasibility and accessibility, particularly in remote locations.

### Limitations

Although a broad and comprehensive search of key databases was conducted, a perceived limitation of this study may have been the exclusion of the database CINAHL. CINAHL was excluded from the search strategy because it predominantly covers allied health practitioners. In addition, participant numbers were small in some of the articles included, in particular qualitative studies that used interviews and focus groups, which may have been due to data saturation. Lastly, convenience sampling was utilised in the majority of studies, which is common in this line of research.

## Conclusions

This review has identified several key challenges that influence the provision of nutrition advice to patients by primary health care providers in Australia and New Zealand, including time constraints, insufficient education and training and associated factors such as low knowledge, confidence and low prioritisation of nutrition. There is clearly scope for improving medical education curricula in the area of nutrition counselling. Primary healthcare education needs to include curricula on evidence-based nutrition that can be implemented effectively within the time constraints of a standard consultation to allow adequate patient counselling in nutrition. This would also require further research to investigate whether brief dietary interventions with patients are indeed effective in improving patients’ dietary habits and nutritional status. Lastly, further research should investigate the perspectives of dentists and pharmacists toward nutrition counselling. 

### Conflict of Interest

The authors declare that they have no conflict of interest. 

## Supplementary material

Supplementary file 1 Appendix 1. PRISMA flow diagram of literature search method
