# Supplementary figures and images for: A review of primary healthcare practitioners’ views about nutrition: implications for medical education
Source: Int J Med Educ. 2022 May 26;13:124–37. doi: 10.5116/ijme.6271.3aa2 (PMC9902177; doi:10.5116/ijme.6271.3aa2)

## Appendix 1

PRISMA flow diagram of literature search method

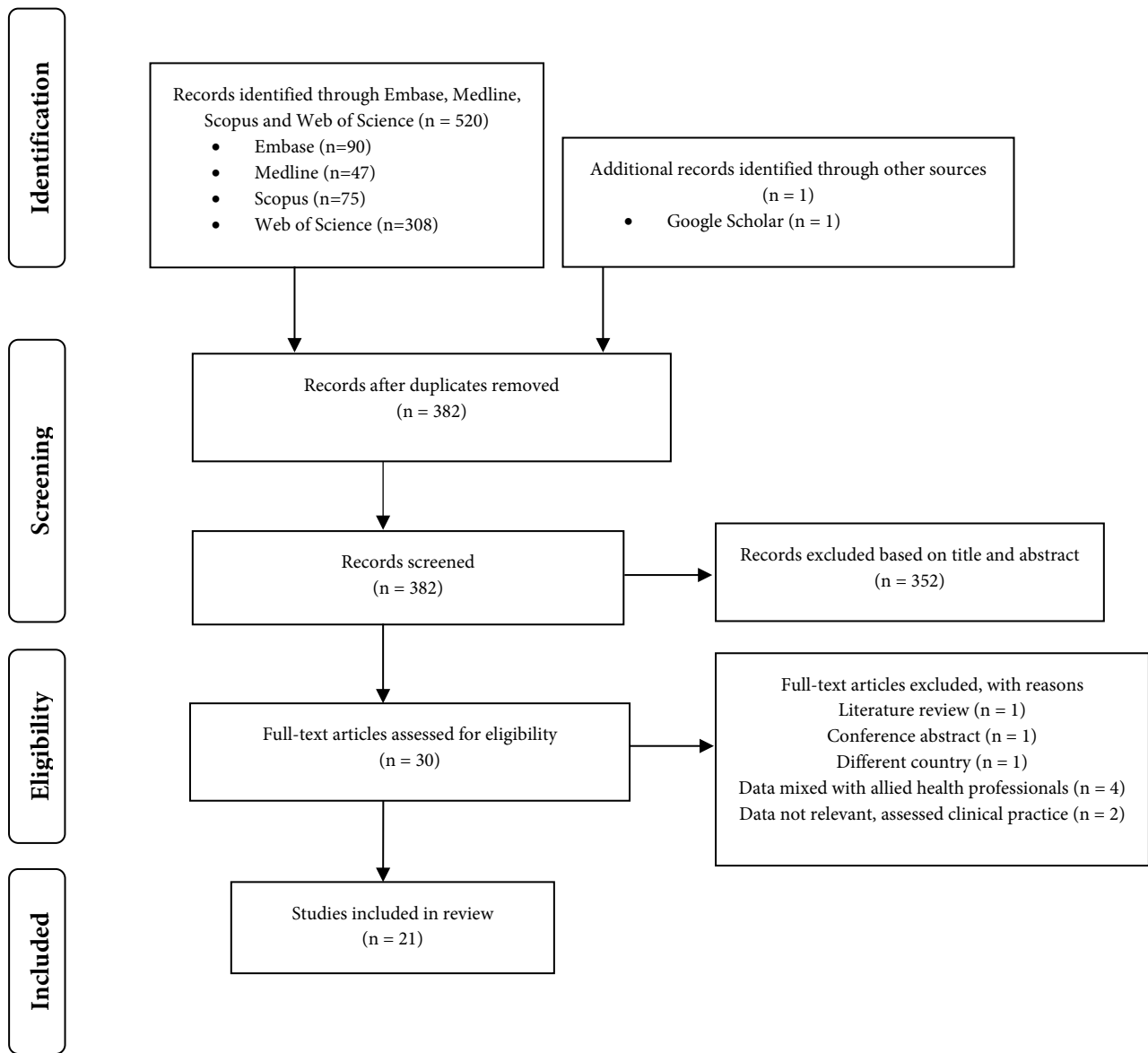

Supplement: Supplementary file 1 — Appendix 1. PRISMA flow diagram of literature search method [file ijme-13-124-S1.pdf]
